# Correction: Preparation of glycerol monostearate from glycerol carbonate and stearic acid

**DOI:** 10.1039/c8ra90049c

**Published:** 2018-06-07

**Authors:** Li Han, Tao Wang

**Affiliations:** State Key Laboratory of Chemical Engineering, Department of Chemical Engineering, Tsinghua University Beijing 100084 China taowang@tsinghua.edu.cn +86 10 62784877 +86 10 62784877

## Abstract

Correction for ‘Preparation of glycerol monostearate from glycerol carbonate and stearic acid’ by Li Han *et al.*, *RSC Adv.*, 2016, **6**, 34137–34145.

The authors regret that Fig. 6 in the original article was incorrect. The caption referred to ^13^C NMR spectra, whereas the figure itself was an expanded version of the ^1^H NMR shown in Fig. 5. The correct version of Fig. 6 is presented below.

**Fig. 6:**
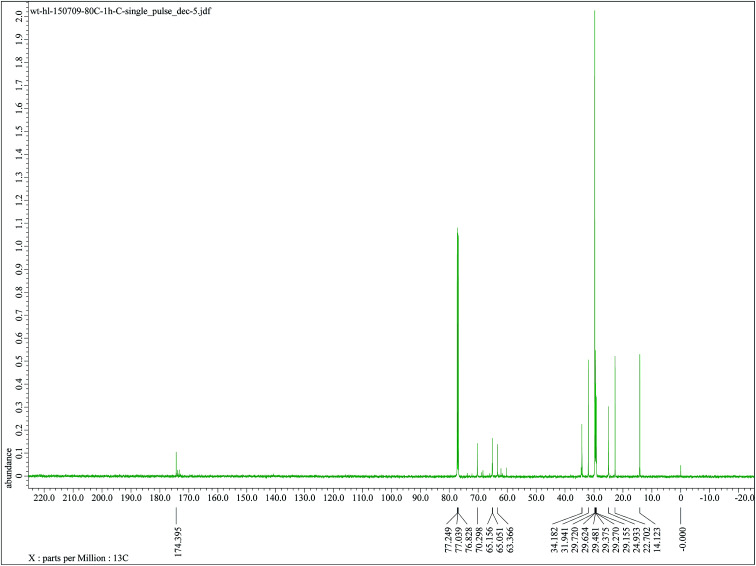
^13^C NMR spectra of GMS.

The Royal Society of Chemistry apologises for these errors and any consequent inconvenience to authors and readers.

## Supplementary Material

